# Spatial and Temporal Expression Patterns of EDA2R, PCDH9, and TRAF7 in *Yotari* (*Dab1^−/−^*) Mice: Implicationsfor Understanding CAKUT Pathogenesis

**DOI:** 10.3390/ijms26136421

**Published:** 2025-07-03

**Authors:** Jelena Komić, Nela Kelam, Anita Racetin, Natalija Filipović, Mirna Saraga-Babić, Dai Ihara, Yu Katsuyama, Katarina Vukojević

**Affiliations:** 1 Department of Family Medicine, Split-Dalmatia County Health Center, 21000 Split, Croatia; jelena.kelam@dz-sdz.hr; 2 Department of Anatomy, Histology and Embryology, University of Split School of Medicine, Šoltanska 2A, 21000 Split, Croatia; nela.kelam@mefst.hr (N.K.); amuic@mefst.hr (A.R.); natalija.filipovic@mefst.hr (N.F.); katarina.vukojevic@mefst.hr (K.V.); 3 Department of Anatomy, Shiga University of Medical Science, Otsu 520-2192, Japan; daiihara@belle.shiga-med.ac.jp (D.I.); kats@belle.shiga-med.ac.jp (Y.K.); 4 Center for Translational Research in Biomedicine, University of Split School of Medicine, Šoltanska 2A, 21000 Split, Croatia; 5 Mediterranean Institute for Life Sciences, University of Split, Meštrovićevo Šetalište 45, 21000 Split, Croatia

**Keywords:** CAKUT, EDA2R, PCDH9, TRAF7, kidney development, *yotari*

## Abstract

Congenital anomalies of the kidney and urinary tract (CAKUT) are the third most common congenital anomaly and a significant public health concern. It is the predominant cause of chronic renal disease in pediatric populations and the principal reason for kidney replacement therapy in individuals under 20, as well as the fourth leading cause in adults. Five candidate genes, including *EDA2R*, *PCDH9*, and *TRAF7* were identified as potential contributors to CAKUT. These genes had not been previously prioritized in CAKUT research, and our prior studies have demonstrated that the proteins encoded by these candidate genes display dysregulated expression across various CAKUT subgroups. Our research examined the expression patterns of EDA2R, PCDH9, and TRAF7 in *yotari (Dab1^−/−^)* mice at two embryonic stages (E13.5 and E15.5) and two postnatal stages (P4 and P14) to ascertain the potential correlation between Reelin–Dab1 signaling, previously linked to CAKUT phenotypes, and the aforementioned proteins through molecular and morphological analyses. All three observed proteins exhibited the highest area percentage at E13.5, with a trend of decline into postnatal stages, during which specific changes in protein expression were noted between the cortex and medulla of *yotari* mice compared to wild-type mice. For TRAF7, a statistically significant difference in area percentage at E13.5 was observed, indicating a link with Reelin–Dab1 signaling and a potentially critical role in the pathophysiology of CAKUT, also marked by our prior study.

## 1. Introduction

Congenital anomalies of the kidney and urinary tract (CAKUT) include a heterogeneous group of mainly structural defects compromising the kidneys, ureters, bladder, and urethra. The term was introduced by Ishikawa and colleagues in 1998 to unify classifications of renal dysplasia, obstructive uropathies, and other urinary tract malformations [1]. CAKUT represents the most common cause of chronic kidney disease in children, with a reported prevalence of 4–60 per 10,000 births, though variability exists due to differences in diagnostic practices and population demographics [2,3]. For perspective, congenital anomalies affect 2–3% of all births, with CAKUT ranking among the most prevalent types after cardiac defects and genetic syndromes [4]. Recent data from the UK National Health Service (NHS) in 2020 identified CAKUT as the second-leading congenital anomaly, with 23.6 cases per 10,000 live births [5].

Under the 2024 EUROCAT guidelines, CAKUT is classified into eight subgroups (including three new categories): (a) unilateral renal agenesis, (b) bilateral renal agenesis/Potter sequence, (c) multicystic renal dysplasia, (d) congenital hydronephrosis/ureteral obstruction, (e) ectopic/lobulated kidneys, (f) bladder exstrophy/epispadias, (g) posterior urethral valves, and (h) prune belly syndrome [6]. Clinically, CAKUT is a major contributor to kidney failure, accounting for the leading cause of kidney replacement therapy (KRT) in patients under the age of 20, and is the fourth-leading cause in adults [7]. Longitudinal studies further underscore its lifelong impact: a cohort of Jewish military recruits revealed that childhood CAKUT, even with normal kidney function, conferred a 4.2-fold increased risk of end-stage renal disease (ESRD) in adulthood [8].

Kidney development is a tightly regulated process involving ureteric bud induction, mesenchymal-to-epithelial transition, and nephron maturation. The renal system derives from the intermediate mesoderm and evolves through three consecutive, partially overlapping phases: the pronephros, mesonephros, and metanephros. The metanephros, or definitive kidney, represents the ultimate developmental stage, originating from the contact between the ureteric bud and the metanephric mesenchyme, which is derived from the caudal segment of the intermediate mesoderm. The metanephric mesenchyme generates the nephron, the functional excretory unit, whereas the collecting system, comprising collecting ducts, originates from the ureteric bud, an epithelial outgrowth of the mesonephric (Wolffian) duct adjacent to its junction with the cloaca [9,10]. Disruptions to these processes, whether genetic, epigenetic, or environmental, can result in CAKUT [11]. To date, over 50 monogenic causes (autosomal dominant, recessive, or X-linked) and 153 genes linked to syndromic CAKUT have been identified [12,13]. Monogenic mutations account for 14–20% of pediatric CAKUT cases, highlighting the importance of genetic testing in diagnosis and counseling [14]. A significant contributor to interindividual genomic variability occurs from structural modifications known as copy number variations (CNVs), which encompass deletions and duplications that disturb the diploid state of genomic regions. These chromosomal rearrangements demonstrate diverse phenotypic effects, varying from neutral impacts to alterations in adaptive features or pathogenic outcomes [15]. Pathogenic CNVs are increasingly recognized as contributors to the genetic etiology of CAKUT, with studies reporting their detection in up to 10.5% of affected individuals [16,17]. Recent work by Westland et al. identified five candidate gene drivers of the CAKUT phenotype in carriers of pathogenic CNVs. They conducted a genome-wide investigation of CNVs in individuals with CAKUT. A multi-step bioinformatics pipeline was employed to prioritize genes. This method examined the infrequent occurrence of CNVs in control samples, gene intolerance measures (such as haploinsufficiency and residual variation intolerance scores), and expression levels during kidney development, which they verified using public databases and confirmed in embryonic mouse kidneys [18]. *DLG1* and *KIF12* were previously investigated by Veljačić et al. because they were among other identified genes highlighted as high-priority novel candidate genes [19]. Other candidate genes *EDA2R*, *PCDH9*, and *TRAF7* were not previously prioritized in CAKUT research, prompting further investigation into their role. Our prior research demonstrated that proteins encoded by these candidate genes are expressed throughout normal human kidney development but exhibit dysregulated expression in distinct CAKUT subgroups [20].

Ectodysplasin A2 Receptor (EDA2R), part of the TNFR family, specifically interacts with the EDA isoform A2. It is located on chromosome Xq13.1 and is commonly referred to as XEDAR. This receptor mediates the activation of the NF-kappa-B and JNK pathways, likely through its binding to TRAF3 and TRAF6 [21]. The NF-kappa-B signaling pathway plays a complex role in cell survival, proliferation, and differentiation [22]. EDA2R is considered a critical regulator of neural, cardiovascular, and respiratory development [23,24,25]. Mutations in the *EDA* lead to a syndrome known as ectodermal dysplasia, which is characterized by abnormal development of ectodermal features such as hair, teeth, nails, and sweat glands [26].

Protocadherin 9 (PCDH9) is a calcium-dependent protein primarily involved in cell adhesion, significantly contributing to cellular attachment in brain tissues. The encoded protein is involved in cellular signaling at neuronal synaptic connections. PCDH9 is situated on chromosome 13q21.32 [27]. Recent investigations have underscored the potential role of diminished PCDH9 expression in the migration of cancer cells, particularly in melanoma [28]. This highlights that PCDH9 may act as a tumor suppressor gene, with its loss of expression recognized as a critical factor in a considerable percentage of hepatocellular carcinoma patients [29]. In addition to its involvement in carcinogenesis, PCDH9 has been linked to psychiatric conditions, including major depressive disorder. Patients affected with this illness exhibit reduced PCDH9 expression in brain tissue and peripheral blood relative to healthy controls [30].

TNF receptor-associated factor 7 (TRAF7) belongs to the TRAF family of E3 ubiquitin ligases. It functions as a dual-specificity enzyme, promoting both ubiquitination and SUMOylation. Regulating different biological processes, including innate immune signals, inflammatory responses, and apoptotic pathways, plays a crucial role in cellular function. It is situated on chromosome 16p13.3 and demonstrates limited tissue specificity across human organs. Nonetheless, in renal tissues, its expression is comparatively elevated in tubular epithelial cells relative to other structural elements of the kidney, such as glomeruli or interstitial compartments [31]. Emerging evidence has demonstrated a functional link between NF-κB signaling and EMT-inducing transcription factors (e.g., Snail, Slug, Twist), which orchestrate epithelial-to-mesenchymal transition (EMT), a developmental process frequently hijacked during tumorigenesis. Studies across multiple malignancies, including breast carcinoma, prostate adenocarcinoma, renal cell carcinoma, and head and neck squamous cell carcinoma, have demonstrated that NF-κB activation correlates with the upregulation of EMT-inducing transcription factors [32,33,34,35]. However, the molecular mechanisms by which NF-κB signaling orchestrates the expression or activity of these EMT-promoting factors remain incompletely understood.

In our previous investigation of human kidney development, EDA2R, PCDH9, and TRAF7 exhibited distinct yet partially overlapping spatiotemporal expression patterns. All three proteins were prominently expressed in ureteric bud cells during early developmental stages, with little to no staining observed in comma- and S-shaped bodies. EDA2R expression was primarily localized to distal tubules, the loop of Henle, and glomeruli but was absent in collecting tubules. In contrast, PCDH9 and TRAF7 showed strong expression in collecting tubules, with PCDH9 displaying a granular staining pattern and TRAF7 presenting a diffuse cytoplasmic distribution [20].

In dysplastic kidneys, all three markers demonstrated increased expression in dysplastic tubules while maintaining largely conserved localization patterns. Notably, EDA2R and PCDH9 had a higher proportion of positive cells in affected kidneys compared to the controls. Conversely, TRAF7-positive cells were more abundant in control kidneys than in observed CAKUT phenotypes [20].

The extracellular matrix protein Reelin and its downstream effector Dab1 (Disabled homolog 1) are evolutionarily conserved regulators of cellular organization, traditionally studied in neurodevelopment [36]. The appearance of Dab1 and Reelin during fetal kidney development confirms their potentially significant role in the formation of kidney structure or function. High Dab1 expression in distal convoluted tubules (DCTs) implies its regulatory role in tubular formation or function maintenance during development. Reelin is highly expressed in human kidneys at early fetal stages, mostly in the proximal convoluted tubule (PCT), while at later fetal stages and the postnatal period its expression decreases [37]. The *yotari* mice (*Dab1^−/−^*) recapitulate CAKUT phenotypes, including podocyte foot process effacement and the absence of filtration slits—hallmarks of developmental podocyte injury. These structural abnormalities resemble those observed in hypoplastic kidneys, supporting the model’s relevance for studying developmental nephropathy, particularly renal hypoplasia, underscoring the functional interdependence of Reelin–Dab1 signaling in nephron maturation [37]. In addition to morphological similarity, our findings, along with those of other researchers, indicate that critical pathogenetic mechanisms, such as increased apoptosis, impaired autophagy, and dysregulated Wnt signaling, are similarly present in both the yotari model and human CAKUT tissue. These common genetic abnormalities reinforce the idea that the physiological and pathophysiological mechanisms of humans and rodents are comparable. Furthermore, the participation of Reelin–Dab1, Wnt/β-catenin, and Notch as conserved pathways in nephron segmentation, ureteric bud branching, and podocyte maturation underscores the model’s suitability for both developmental research and mechanistic analysis, as well as therapeutic investigation [38,39,40]. Therefore, yotari mice provide functional relevance for the preclinical investigation of targeted interventions and the modeling of human CAKUT pathogenesis, in addition to phenotypic similarity. Our study aims to investigate the regulatory roles of *Dab1* functional silencing on the spatiotemporal expression patterns and subcellular localization of EDA2R, PCDH9, and TRAF7 during embryonic and postnatal renal development in *yotari* mice. By integrating molecular and morphological analyses, we aimed to elucidate how disrupted Reelin–Dab1 signaling influences key pathways implicated in nephrogenesis.

## 2. Results

### 2.1. EDA2R Immunoexpression

On the embryonic day E13.5, the protein was prominently localized to the basolateral membrane of the ureteric bud, with strong granular staining observed in the ampulla. A punctate and diffuse cytoplasmic signal was detected in condensations of the metanephric mesenchyme and renal vesicles of wild-type animals.

In *yotari* mutant animals, intense cytoplasmic staining was observed in renal vesicles and the metanephric mesenchyme, whereas staining in the ampulla and ureteric bud was weaker, punctate, and diffusely distributed throughout the cytoplasm.

**Figure 1:**
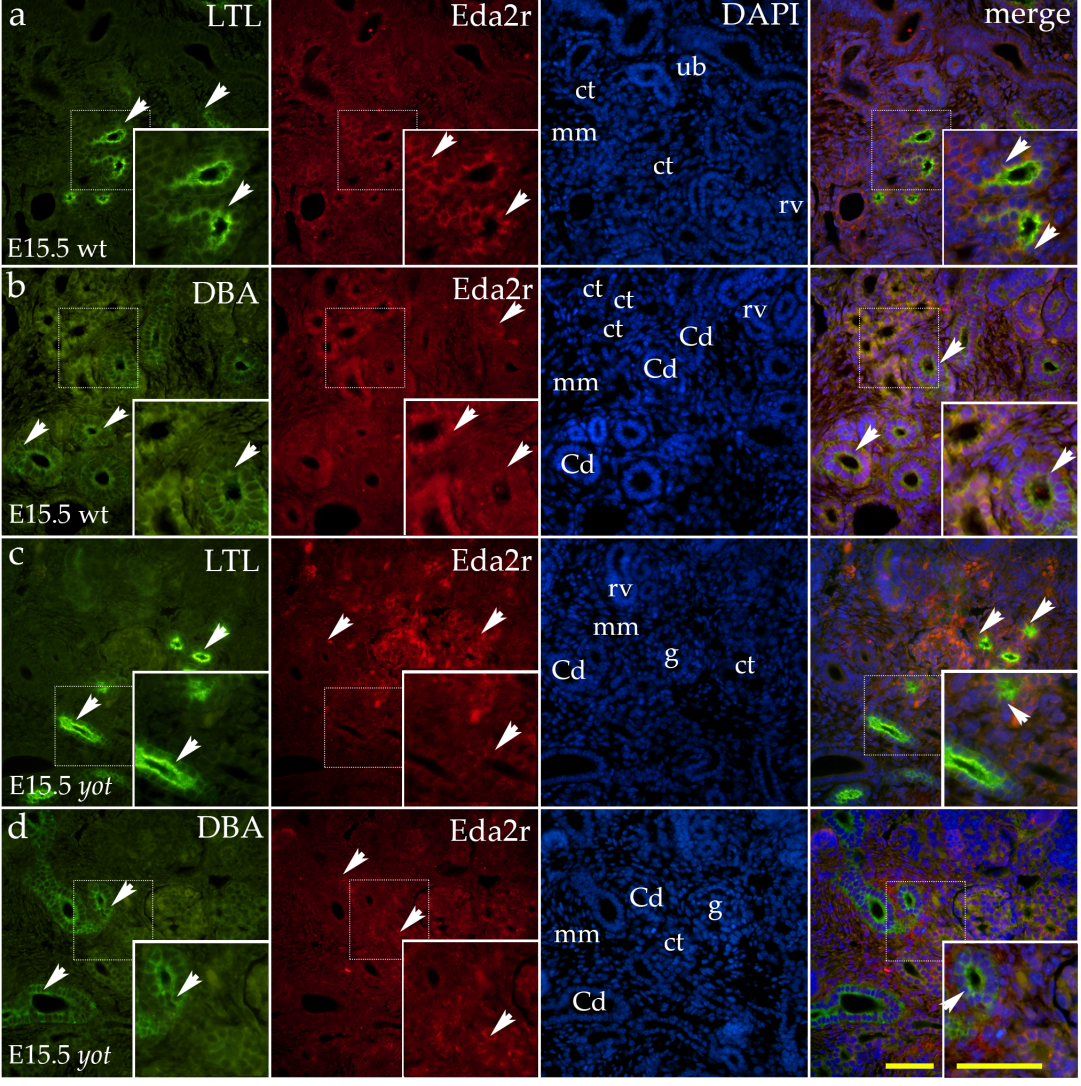
Double immunofluorescence staining of wild-type (wt) and *yotari* (*yot*) mouse kidneys at embryonic day 15.5 (E15.5) highlights Lotus tetragonolobus lectin (LTL), a marker for differentiated proximal tubules ((**a**,**c**); green staining) and Dolichos biflorus agglutinin (DBA), a marker for developing renal collecting ducts ((**b**,**d**); green staining), along with Ectodysplasin A2 Receptor (EDA2R) (**a**–**d**; red staining). At E15.5, both genotypes showed strong diffuse cytoplasmic staining in convoluted tubules as demonstrated in the LTL stained images (**a**,**c**) and weak staining in renal vesicles (**b**). Granular staining persisted in ampullae/ureteric buds at reduced intensity compared to E13.5 and colocalized with DBA (**b**,**d**). White arrows indicate regions of positive fluorescent signal corresponding to LTL, DBA and EDA2R, localized in distinct renal structures, including the metanephric mesenchyme (mm), renal vesicles (RVs), glomeruli (g), convoluted tubules (CTs), ampullae (A), ureteric bud (ub), and collecting ducts (CDs), as visualized in images of 4′,6-diamidino-2-phenylindole (DAPI)-stained nuclei. White arrows in the merged images of LTL, DBA, EDA2R, and DAPI highlight regions of co-expression (orange staining). The inserts that match the dashed boxes indicate the major region of protein expression. Images were captured at 40× magnification, with a scale bar of 50 μm applicable to all images. The images shown represent spatial localization and colocalization with tubular segment-specific markers and may not reflect the average signal intensity.

At E15.5, both genotypes exhibited strong diffuse cytoplasmic staining in convoluted tubules (Figure 1a) and weak staining in renal vesicles (Figure 1b). Granular staining was also present in ampullae, although at a reduced intensity compared to E13.5 (Figure 1b).

**Figure 2:**
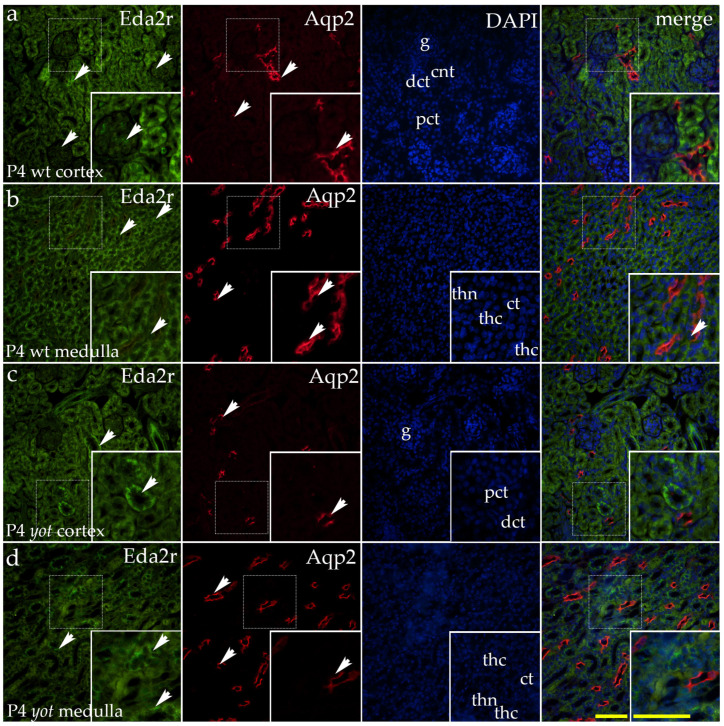
Double immunofluorescence staining of wild-type (wt) and *yotari* (*yot*) mouse kidney cortex (**a**,**c**) and medulla (**b**,**d**) at postnatal day 4 (P4) highlights Ectodysplasin A2 Receptor (EDA2R) ((**a**–**d**); green staining), along with Aquaporin 2 (Aqp2), a marker for principal cells of the collecting ducts and connecting tubules ((**a**–**d**); red staining). In wild-type mice at P4, moderate staining was observed in the visceral layer of Bowman’s capsule and occasionally in the vascular endothelium. Distal tubules showed weak, diffuse cytoplasmic staining, while there was a rare punctate signal in proximal tubules localized to apical or basal membranes (**a**). In the medulla, weak staining was present in thick segments of the loop of Henle, with occasional signals in thin segments and collecting ducts (**b**). *Yotari* exhibited a similar pattern (**c**,**d**), but with markedly increased staining in the thick segments of the loop of Henle (**d**). Minimal co-localization with Aqp2 was detected (**b**). White arrows indicate regions of positive fluorescent signal corresponding to EDA2R and Aqp2 in various renal structures, including glomeruli (g), proximal convoluted tubules (PCTs), distal convoluted tubules (DCTs), connecting tubules (CNTs) of the renal cortex, and the thin (thn) and thick (thc) segments of the loop of Henle and collecting tubules (CTs) of the renal medulla, as visualized in images of 4′,6-diamidino-2-phenylindole (DAPI)-stained nuclei. White arrows in the merged images of EDA2R, Aqp2, and DAPI highlight regions of co-expression (orange staining). The inserts that match the dashed boxes indicate the major region of protein expression. Images were captured at 40× magnification, with a scale bar of 50 μm applicable to all images. The images shown represent spatial localization and colocalization with tubular segment-specific markers and may not reflect the average signal intensity.

In the postnatal period, moderate staining was detected in the visceral layer of Bowman’s capsule and sporadically in the vascular endothelium (Figure 2a). Staining in distal convoluted tubules was weak, diffuse, and cytoplasmic (Figure 2a). In proximal convoluted tubules, the signal was rare but, where present, appeared punctate and localized to either the apical or basal membrane of the tubules (Figure 2a). The renal medulla displayed weak staining in the thick segments of the loop of Henle, with occasional positive cells in the thin segments (Figure 2b). Scarce signal was detected in the cells of the conducting system, including the collecting ducts (Figure 2b). In *yotari* mutants, a similar staining pattern was observed (Figure 2c,d), with a notable distinction: strong EDA2R expression was detected in the renal medulla, particularly in the thick segments of the loop of Henle (Figure 2d).

**Figure 3:**
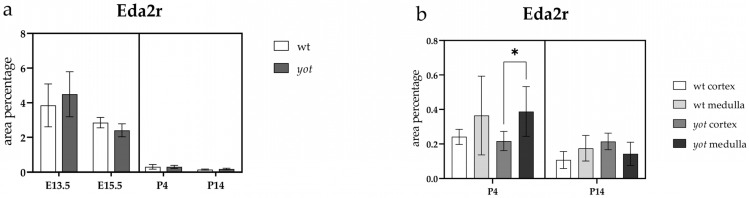
Quantification of Ectodysplasin A2 Receptor (EDA2R) expression in wild-type (wt) and *yotari* (*yot*) mouse kidneys at embryonic days 13.5 (E13.5) and 15.5 (E15.5) and postnatal days 4 (P4) and 14 (P14) (**a**). EDA2R expression area percentages in the cortex and medulla of wild-type and *yotari* kidneys at P4 and P14 (**b**). The data are presented as the mean ± SD (vertical lines) and analyzed using a two-way ANOVA followed by Tukey’s multiple comparison test. For each developmental stage, ten representative images were analyzed per region. The results were averaged per examined group. Significant difference was indicated by * *p* < 0.05.

We did not observe a significant difference in the area percentage of EDA2R-positive cells between the examined animal phenotypes across all studied embryonic and postnatal developmental stages (Figure 3a).

At P4, we detected a statistically significant difference in EDA2R expression between the cortex and medulla of the *yotari* phenotype, following a similar trend as in wild-type animals but without statistical significance (Figure 3b).

At P14, EDA2R expression exhibited only minor variations between the cortex and medulla in both examined animal phenotypes (Figure 3b).

### 2.2. PCDH9 Immunoexpression

**Figure 4:**
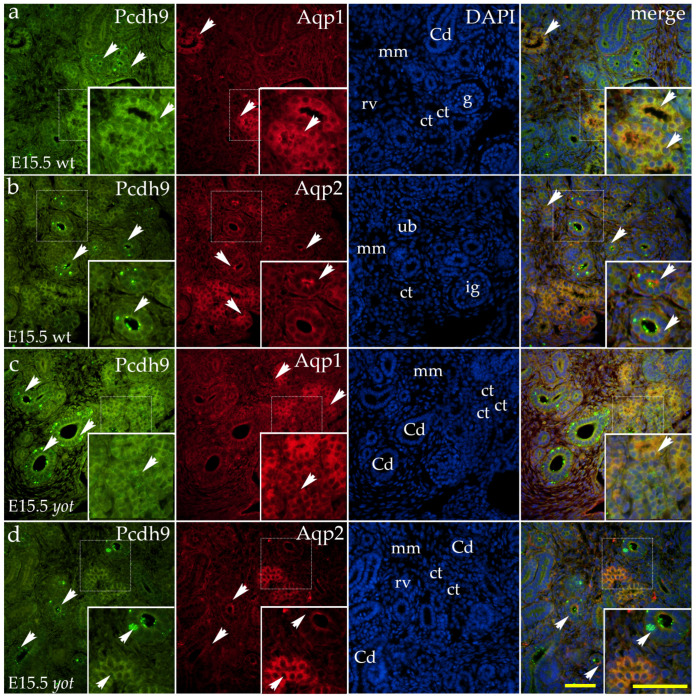
Double immunofluorescence staining of wild-type (wt) and *yotari* (*yot*) mouse kidneys at embryonic day 15.5 (E15.5) highlights Protocadherin 9 (PCDH9) ((**a**–**d**); green staining), along with Aquaporin 1 (Aqp1), which labels the apical and basolateral membranes of proximal tubule cells (**a**,**c**; red staining) and Aquaporin 2 (Aqp2), a marker for principal cells in developing collecting ducts and ureteric bud derivatives ((**b**,**d**); red staining). White arrows indicate regions of positive fluorescent signal corresponding to PCDH9, Aqp1, and Aqp2 in various renal structures, including the metanephric mesenchyme (mm), renal vesicles (RVs), glomeruli (g), convoluted tubules (CTs), ampullae (A), ureteric bud (ub), immature glomeruli (ig), and collecting ducts (CDs), as visualized in images of 4′,6-diamidino-2-phenylindole (DAPI)-stained nuclei. Strong, coarse granular staining is localized to the basolateral membranes of ureteric bud ampullae and collecting ducts (**a**–**d**). In proximal convoluted tubules, PCDH9 appears as granular staining on the apical membrane (**a**,**c**), while weaker, diffuse membrane staining is observed in other developing convoluted tubules (**a**,**d**). White arrows in the merged images of PCDH9, Aqp1, Aqp2, and DAPI highlight regions of co-expression (orange staining). The inserts that match the dashed boxes indicate the major region of protein expression. Images were captured at 40× magnification, with a scale bar of 50 μm applicable to all images. The images shown represent spatial localization and colocalization with tubular segment-specific markers and may not reflect the average signal intensity.

PCDH9 shows a characteristic expression pattern during kidney development. A strong, coarse granular signal is present on the basolateral membranes of the ureteric bud ampullae and collecting ducts (Figure 4a–d). In the proximal convoluted tubule, expression appears as granular staining on the apical membrane (Figure 4a,c), whereas in the remaining developing convoluted tubules, weaker diffuse staining is observed along the membranes (Figure 4a,d).

**Figure 5:**
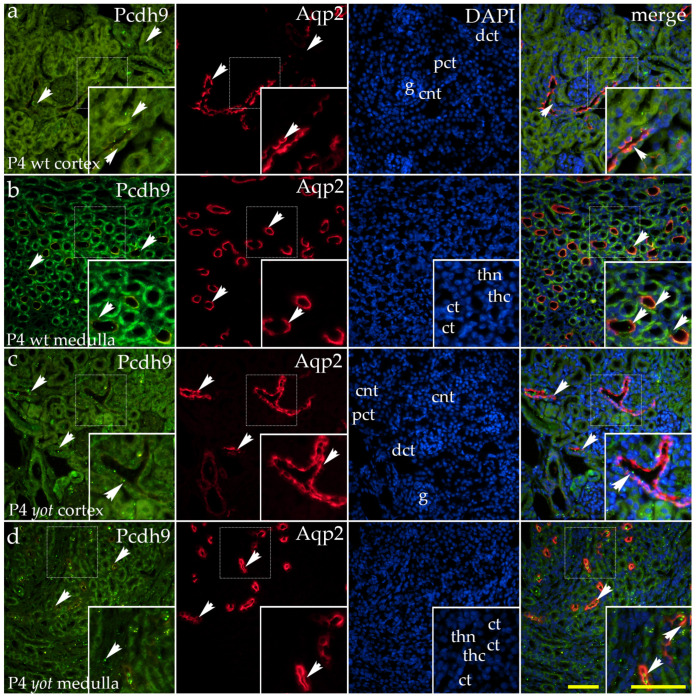
Double immunofluorescence staining of wild-type (wt) and *yotari* (*yot*) and mouse kidney cortex (**a**,**c**) and medulla (**b**,**d**) at postnatal day 4 (P4) highlights Protocadherin 9 (PCDH9) ((**a**–**d**); green staining), along with Aquaporin 2 (Aqp2), a marker for principal cells of the collecting ducts and connecting tubules ((**a**–**d**); red staining). In wild-type mice, diffuse cytoplasmic staining was observed in the visceral layer of Bowman’s capsule, with a strong punctate signal on the apical membranes of connecting tubules (**a**). Distal convoluted tubules showed diffuse cytoplasmic staining, most intense basolaterally, while a punctate signal was seen in the vascular endothelium. Proximal tubules showed a randomly distributed signal. In the renal medulla, strong granular staining was detected on the apical membranes of collecting ducts; thick segments of Henle’s loop showed diffuse staining, with no signal in thin segments (**b**). *Yotari* exhibited a similar spatial pattern with markedly stronger signals. Larger apical granules in connecting tubules and diffuse distal tubule staining were observed, possibly indicating membrane microdomains, vesicles, or endosomal compartments involved in PCDH9 trafficking (**c**). In the medulla, *yotari* mirrored wild-type expression (**d**). White arrows indicate regions of positive fluorescent signal corresponding to PCDH9 and Aqp2 in various renal structures, including glomeruli (g), proximal convoluted tubules (PCTs), distal convoluted tubules (DCTs), connecting tubules (CNTs) of the renal cortex, and the thin (thn) and thick (thc) segments of the loop of Henle and collecting tubules (CTs) of the renal medulla, as visualized in images of 4′,6-diamidino-2-phenylindole (DAPI)-stained nuclei. White arrows in the merged images of PCDH9, Aqp2, and DAPI highlight regions of co-expression (orange staining). The inserts that match the dashed boxes indicate the major region of protein expression. Images were captured at 40× magnification, with a scale bar of 50 μm applicable to all images. The images shown represent spatial localization and colocalization with tubular segment-specific markers and may not reflect the average signal intensity quantified in Figure 6.

During the postnatal development of wild-type mice, diffuse cytoplasmic staining was observed in the visceral layer of the Bowman’s capsule (Figure 5a). Additionally, a strong punctate signal was detected on the apical membranes of the connecting tubules (Figure 5a). Diffuse cytoplasmic staining was also present in the distal convoluted tubules, with the most intense signal localized to their basolateral compartments. Punctate staining was observed in the vascular endothelium, while the signal in the proximal convoluted tubules appeared randomly distributed (Figure 5a).

In the renal medulla of wild-type mice, a pronounced granular signal was detected on the apical membranes of the collecting ducts. The thick segments of Henle’s loop exhibited diffuse cytoplasmic staining, whereas the thin segments lacked detectable signals (Figure 5b).

The *yotari* mutant exhibited a similar spatial expression pattern to the investigated protein but with a markedly stronger signal. Larger accumulations of PCDH9 signals in the form of granules were observed on the apical membranes of the connecting tubules, along with diffuse staining of the distal convoluted tubules (Figure 5c). In the renal medulla of *yotari* mutants, the staining pattern mirrored that of wt mice, displaying a granular signal on the apical membranes of the collecting ducts, diffuse staining in the thick segments of Henle’s loop, and an absence of signal in the thin segments of Henle’s loop (Figure 5d).

**Figure 6:**
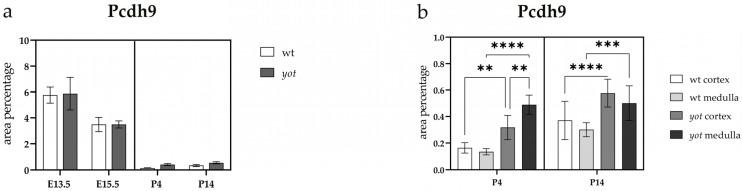
Quantification of Protocadherin 9 (PCDH9) expression in wild-type (wt) and *yotari* (*yot*) mouse kidneys at embryonic days 13.5 (E13.5) and 15.5 (E15.5) and postnatal days 4 (P4) and 14 (P14) (**a**). PCDH9 expression area percentages in the cortex and medulla of wild-type and *yotari* kidneys at P4 and P14 (**b**). The “area percentage” refers to the proportion of the image occupied by a positive fluorescent signal, calculated as the number of fluorescent pixels above a defined threshold divided by the total number of pixels in the image. The data are presented as the mean ± SD (vertical lines) and analyzed using a two-way ANOVA followed by Tukey’s multiple comparison test. For each developmental stage, ten representative images were analyzed per region. The results were averaged per examined group. Significant differences were indicated by ** *p* < 0.001, *** *p* < 0.0001, and **** *p* < 0.00001.

The area percentage occupied by the PCDH9-positive signal was consistent, with no significant differences observed between wild-type (wt) and *yotari* (*yot*) animals across all examined embryonic and postnatal developmental stages (Figure 6a).

In both analyzed postnatal stages, P4 and P14, a statistically significant increase in PCDH9 expression was observed in the cortex and medulla of *yotari* animals compared to wt animals (Figure 6a).

### 2.3. TRAF7 Immunoexpression

**Figure 7:**
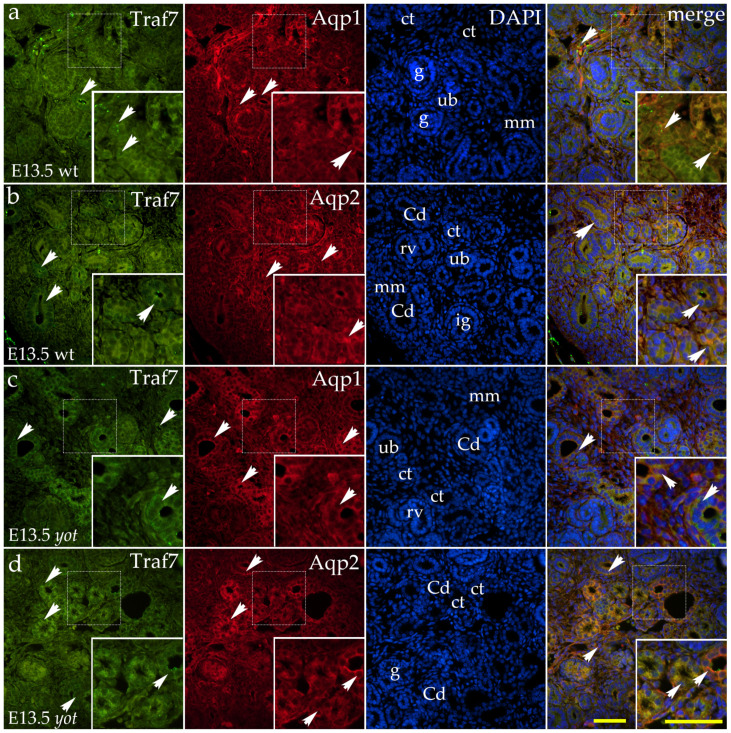
Double immunofluorescence staining of *yotari* (*yot*) and wild-type (wt) mouse kidneys at embryonic day 13.5 (E13.5) highlights TNF receptor-associated factor 7 (TRAF7) ((**a**–**d**)**;** green staining), along with Aquaporin 1 (Aqp1), which labels the apical and basolateral membranes of proximal tubule cells ((**a**,**c**); red staining) and Aquaporin 2 (Aqp2), a marker for principal cells in developing collecting ducts and ureteric bud derivatives ((**b**,**d**); red staining). In wild-type embryos, strong punctate staining was observed on the basolateral and apical membranes of the ampullae, ureteric bud, and collecting ducts (**a**,**b**). A mild diffuse cytoplasmic signal was detected in renal vesicles, immature glomeruli, and metanephric mesenchyme (**a**). In *yotari*, membrane-associated punctate staining remained strong in the ampullae and ureteric bud (**c**), while diffuse cytoplasmic staining in renal vesicles, immature glomeruli, and the ureteric bud was reduced (**c**,**d**). White arrows indicate regions of the positive fluorescent signal corresponding to TRAF7, Aqp1, and Aqp2 in various renal structures, including the metanephric mesenchyme (mm), renal vesicles (RVs), glomeruli (g), convoluted tubules (CTs), ampullae (A), immature glomeruli (ig), ureteric bud (ub), and collecting ducts (CDs), as visualized in images of 4′,6-diamidino-2-phenylindole (DAPI)-stained nuclei. White arrows in the merged images of TRAF7, Aqp1, Aqp2, and DAPI highlight regions of co-expression (orange staining). The inserts that match the dashed boxes indicate the major region of protein expression. Images were captured at 40× magnification, with a scale bar of 50 μm applicable to all images. The images shown represent spatial localization and colocalization with tubular segment-specific markers and may not reflect the average signal intensity.

At embryonic day 13.5, strong punctate staining was observed on the basolateral and apical membranes of the ampullae, ureteric bud, and collecting ducts (Figure 7a,b). A mild diffuse green signal was detected in the cytoplasm of renal vesicles, immature glomeruli, and the metanephric mesenchyme (Figure 7a).

In *yotari* mutant animals, the staining pattern remained similar, with strong punctate signals on the ampullae and ureteric bud membranes (Figure 7c). However, the diffuse cytoplasmic staining in renal vesicle/immature glomerular cells (Figure 7c) and the ureteric bud was notably weaker (Figure 7d).

By E15.5, a comparable staining pattern was observed, with additional moderate to strong punctate cytoplasmic staining in the convoluted tubules. In *yotari* mutants, the staining intensity in these structures was more pronounced.

**Figure 8:**
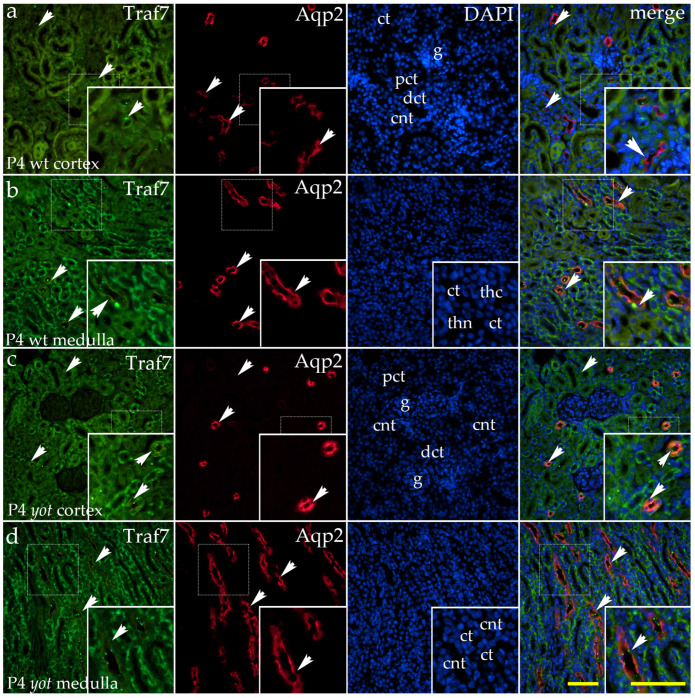
Double immunofluorescence staining of wild-type (wt) and *yotari* (*yot*) and mouse kidney cortex (**a**,**c**) and medulla (**b**,**d**) at postnatal day 4 (P4) highlights TNF receptor-associated factor 7 (TRAF7) ((**a**–**d**); green staining), along with Aquaporin 2 (Aqp2), a marker for principal cells of the collecting ducts and connecting tubules ((**a**–**d**); red staining). White arrows indicate regions of the positive fluorescent signal corresponding to TRAF7 and Aqp2 in various renal structures, including glomeruli (g), proximal convoluted tubules (PCTs), distal convoluted tubules (DCTs), connecting tubules (CNTs) of the renal cortex, and the thin (thn) and thick (thc) segments of the loop of Henle and collecting tubules (CTs) of the renal medulla, as visualized in images of 4′,6-diamidino-2-phenylindole (DAPI)-stained nuclei. In wild-type mice, mild diffuse cytoplasmic staining was observed in glomeruli, with a moderate diffuse signal in distal convoluted tubules and strong granular staining on the apical membranes of connecting tubules (**a**). In the medulla, moderate diffuse staining was present in the thick segments of the loop of Henle, mild staining in collecting tubules, and strong granular apical staining in connecting tubules; no signal was detected in thin segments (**b**). In *yotari*, distal tubules exhibited stronger staining with randomly distributed puncta on apical membranes (**c**). Glomerular staining remained weak, and proximal tubules lacked a detectable signal. In the medulla, strong staining persisted in the thick segments of Henle’s loop, while thin segments and collecting tubules showed no staining (**d**). White arrows in the merged images of TRAF7, Aqp2, and DAPI highlight regions of co-expression (orange staining). The inserts that match the dashed boxes indicate the major region of protein expression. Images were captured at 40× magnification, with a scale bar of 50 μm applicable to all images. The images shown represent spatial localization and colocalization with tubular segment-specific markers and may not reflect the average signal intensity.

At P4, mild diffuse cytoplasmic staining was detected in glomeruli while moderate diffuse staining was present in distal convoluted tubules. A strong, granular signal was localized to the apical membranes of the connecting tubules (Figure 8a). The medulla of the wild-type animals demonstrated moderate diffuse cytoplasmatic staining in the thick portions of the loop of Henle, mild staining in collecting tubules, and strong, granular staining of the apical membranes of the connecting tubules. The thin segments of the loop of Henle lacked the positive signal (Figure 8b).

In *yotari* mutants, the staining intensity in the distal convoluted tubules was elevated, showing randomly distributed strong punctate expression on the apical membrane of the tubules (Figure 8c). The staining in the glomeruli remained weak, and the proximal convoluted tubules lacked detectable signals (Figure 8c). In the kidney medulla, strong staining was present in the thick segment of the loop of Henle, while the thin segments and collecting tubules were devoid of staining (Figure 8d).

**Figure 9:**
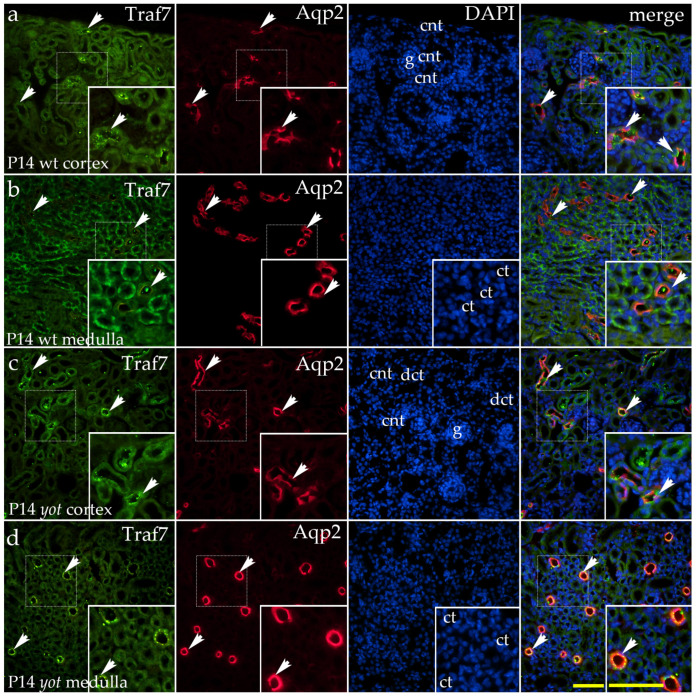
Double immunofluorescence staining of wild-type (wt) and *yotari* (*yot*) and mouse kidney cortex (**a**,**c**) and medulla (**b**,**d**) at postnatal day 14 (P14) highlights TNF receptor-associated factor 7 (TRAF7) ((**a**–**d**); green staining), along with Aquaporin 2 (Aqp2), a marker for principal cells of the collecting ducts and connecting tubules ((**a**–**d**); red staining). White arrows indicate regions of positive fluorescent signal corresponding to TRAF7 and Aqp2 in various renal structures, including glomeruli (g), proximal convoluted tubules (PCTs), distal convoluted tubules (DCTs), connecting tubules (CNTs) of the renal cortex, and the thin (thn) and thick (thc) segments of the loop of Henle and collecting tubules (CTs) of the renal medulla, as visualized in images of 4′,6-diamidino-2-phenylindole (DAPI)-stained nuclei. In wild-type mice, the staining pattern resembled that observed at P4 (**a**,**b**). In yotari mutants, strong punctate staining appeared on the apical membrane of proximal convoluted tubules (**c**). In the medulla, staining was present in both thin and thick segments of the loop of Henle while collecting tubules remained unstained (**d**). White arrows in the merged images of TRAF7, Aqp2, and DAPI highlight regions of co-expression (orange staining). The inserts that match the dashed boxes indicate the major region of protein expression. Images were captured at 40× magnification, with a scale bar of 50 μm applicable to all images. The images shown represent spatial localization and colocalization with tubular segment-specific markers and may not reflect the average signal intensity quantified in Figure 10.

By P14, the staining pattern was similar to that observed at P4 (Figure 9a–d). However, in *yotari* mutants, strong punctate staining was also found on the apical membrane of the proximal convoluted tubules (Figure 9c). Additionally, in the medulla, staining was detected in both the thin and thick segments of the loop of Henle, whereas the collecting tubules remained unstained (Figure 9d).

**Figure 10:**
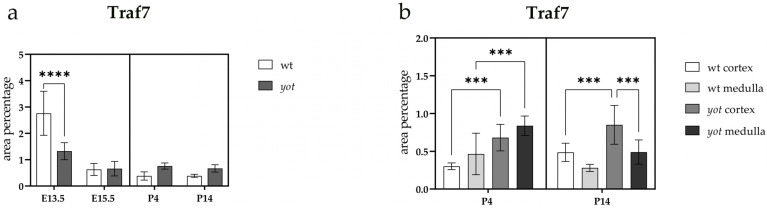
Quantification of TNF receptor-associated factor 7 (TRAF7) expression in wild-type (wt) and *yotari* (*yot*) mouse kidneys at embryonic days 13.5 (E13.5) and 15.5 (E15.5) and postnatal days 4 (P4) and 14 (P14) (**a**). TRAF7 expression area percentages in the cortex and medulla of wild-type and *yotari* kidneys at P4 and P14 (**b**). The “area percentage” refers to the proportion of the image occupied by a positive fluorescent signal, calculated as the number of fluorescent pixels above a defined threshold divided by the total number of pixels in the image. The data are presented as the mean ± SD (vertical lines) and analyzed using a two-way ANOVA followed by Tukey’s multiple comparison test. For each developmental stage, ten representative images were analyzed per region. The results were averaged per examined group. Significant differences were indicated *** *p* < 0.0001, and **** *p* < 0.00001.

At E13.5, a statistically significant reduction in the area percentage covered by the TRAF7-positive signal was observed in *yotari* animals compared to wild-type animals (wt) (*p* < 0.0001) (Figure 10a). At E15.5 and throughout the postnatal period, no statistically significant differences were detected between the phenotypes examined (Figure 10a). At P4, TRAF7 expression was significantly higher in the cortex and medulla of *yotari* animals compared to wt animals (*p* < 0.001) (Figure 10b). At P14, TRAF7 expression in the medulla was significantly lower than in the cortex in *yotari* animals (*p* < 0.001), following a similar trend in wt animals, although without statistical significance (Figure 10b). Additionally, a statistically significant reduction in the percentage of the TRAF7-positive area was noted in the renal cortex of wt animals compared to the *yotari* phenotype (Figure 10b).

## 3. Discussion

CAKUT represents a significant public health issue and is the main cause of chronic kidney disease in pediatric populations. It is crucial to implement early intervention techniques to postpone the initiation of renal replacement therapy (RRT) and unraveling the genetic and molecular pathways generating CAKUT is of the greatest importance. This study aims to investigate the relationship between the functional silence of *Dab1* and three candidate genes—*EDA2R*, *PCDH9*, and *TRAF7*—to assess their possible role in the signaling pathways associated with the Reelin–Dab1 axis. Our objective was to determine if these proteins engage with Reelin–Dab1 signaling to facilitate renal hypoplasia and influence the development of CAKUT.

Our research did not find significant differences in the expression of EDA2R between the examined animal phenotypes across all investigated embryonic and postnatal developmental stages. However, at P4, a statistically significant difference in EDA2R expression was detected between the cortex and medulla in *yotari* mice, with a similar trend noted in wild-type (wt) animals, although it did not reach statistical significance. The aforementioned observation aligns with our prior studies made on human embryonic specimens, which similarly reported no significant differences between the two renal compartments at the Ph3 stage of kidney development, which is equivalent to P4 in mice [20,41,42,43]. Nephrogenesis in mice begins around E11.5, corresponding to the initiation of Ph1 (i.e., week 5) in humans. E15.5 corresponds to week 15 of human renal development, signifying the conclusion of developmental Ph1. The advancement of nephrons and renal structures persists during Ph2 and Ph3. By the conclusion of Ph3, i.e., at the onset of Ph4, which aligns with week 36 in humans and P4 in mice, the formation of new nephrons is finalized [41,42]. Given the uniform expression of EDA2R between the two animal genotypes, as well as between individual renal compartments, we can conclude that the conditional *Dab1^−/−^* mutation does not influence EDA2R expression. This suggests that EDA2R is regulated independently through alternative signaling pathways. The kidney abnormalities observed in *yotari* mice, such as hypoplasia, foot process effacement, and absent filtration slits, are characteristic features of developmental podocyte injury. These changes, which are distinctive hallmarks of hypoplastic kidneys, arise as part of the developmental process underlying podocyte dysfunction. Lan et al. showed that the overexpression of EDA2R due to high glucose milieu led to podocyte injury and dedifferentiation [44]. Although both mechanisms of kidney dysfunction are podocyte injuries, the following facts can lead to the conclusion that the absence of EDA2R upregulation in *yotari* mice suggests its role is context-dependent and limited to acquired metabolic injury rather than developmental pathways.

The percentage of area occupied by a PCDH9 positive signal was consistent across all embryonic and postnatal developmental stages examined, with no significant differences observed between wild-type and *yotari* mice. Also, the expression of PCDH9 was higher in the embryonic phase than postnatal. This expression trend aligns with our prior research, which documented a negative correlation between PCDH9 expression and developmental progression in healthy human embryonic and postnatal kidneys [20]. The elevated expression of PCDH9 during embryonic stages, coupled with its declining trend during postnatal development, suggests that PCDH9 plays a more critical role in EMT processes during renal morphogenesis rather than in maintaining renal function postnatally.

In both postnatal stages examined, statistically significant upregulation of PCDH9 expression was observed in the renal cortex and medulla of *yotari* mice compared to wild-type controls. This finding is partially consistent with our prior research on developing human kidneys, which demonstrated statistically significant downregulation of PCDH9 protein expression in the renal cortex relative to the medulla during developmental phase 3 (equivalent to mouse P4 kidneys). However, no significant differences in PCDH9 expression were observed in developmental phase 4 (corresponding to mouse P14 kidneys) [20,42,43].

An increase in PCDH9 expression in the cortex and medulla of *yotari* mice at postnatal stages P4 and P14, relative to wild-type mice, may indicate a compensatory mechanism for impaired kidney development due to *Dab1* deficiency. The *Dab1* mutation in *yotari* mice disrupts Reelin signaling, resulting in renal hypoplasia [38]. PCDH9 is associated with cell–cell adhesion and cytoskeletal organization and may be upregulated to address structural and functional defects during nephrogenesis [20,28]. This hypothesis aligns with studies demonstrating that reduced fibroblast growth factor receptor (FGFR1/FGFR2) expression in *yotari* kidneys contributes to impaired nephron development, while extracellular signal-regulated kinase (Erk1/2) and mammalian target of rapamycin (mTOR) pathways are downregulated, further exacerbating developmental abnormalities [45]. Increased expression of PCDH9 may indicate an adaptive response to increased autophagic activity noted in *yotari* kidneys, as evidenced by the upregulation of autophagy markers like LC3B and LAMP2A during postnatal development [39]. PCDH9 downregulation is associated with enhanced cell migration in human CAKUT models; conversely, its overexpression in *yotari* mice may stabilize cell–cell contacts and maintain tissue integrity during developmental stress. The findings indicate that PCDH9 has a context-dependent role in kidney development and pathology, likely influencing cellular responses to altered signaling pathways.

We have observed a substantial decrease in TRAF7 expression at E13.5 in *yotari* (*Dab1*^−/−^) mice compared to wild-type controls. This suggests that the *Dab1* mutation has an early-stage effect on TRAF7 expression. Previous research indicates that TRAF7 is linked to many developmental anomalies, including cardiac looping defects and other cardiac, craniofacial, and ciliary abnormalities, which in the setting of the kidneys manifested as kidney cysts and polycystic kidney disease [46,47]. An essential determinant of successful nephrogenesis is efficient vasculogenesis, which is integral to the formation of all renal structures and exhibits significant spatial and temporal complexity [48]. Kidney vascularization commences at E11, and by E13.5, vascular plexuses develop in designated places (e.g., nephrogenic zones), while pre-existing endothelial networks differentiate into distinct arteries in other areas [49,50]. Titsikov et al. emphasized the crucial function of *TRAF7* in endothelial development [51]. They demonstrated that by E10, *TRAF7^−/−^* embryos perished as a result of impaired blood vessel integrity, which led to intraembryonic hemorrhage and hypoxia. Additionally, it has been demonstrated that Reelin, whose signaling pathway is known to be associated with the Dab1 protein, regulates the integrity of the blood vessel wall by affecting various processes critical for its development, including endothelial cell adhesion, morphology, as well as membrane resistance and permeability [52,53]. Accordingly, it can be assumed that TRAF7 is a possible novel cascade factor of the Reelin signaling pathway.

Additionally, our prior research demonstrated the largest area percentage of TRAF7 specifically during phase 1 development of the control human samples [20]. Phase 1 of human kidney development, spanning gestational weeks 5 to 14, aligns with embryonic weeks E11.5 to E15.5, and peak TRAF7 expression in our current study was observed at E13.5, the midpoint of this stage [42,43]. By E15.5, TRAF7 expression levels in *yotari* mice reverted to comparable levels as in wild-type controls, exhibiting a general decline in both groups that persisted throughout the postnatal period, suggesting that the impact of *Dab1* functional silencing on TRAF7 expression is stage-specific. Our prior research similarly demonstrated a pattern of diminished TRAF7 expression across the developmental phases in comparison to the initial phase 1 [20].

Moreover, our research group has previously identified reduced TRAF7 expression in four examined CAKUT subtypes, including the hypoplastic kidney. We demonstrated that the hypoplastic kidney had a significantly lower area percentage of TRAF7 than the control human embryonic and fetal kidneys [20]. Racetin et al. demonstrated that hypoplastic kidneys are a distinctive phenotype in *yotari* mice, which we found in the current study to have a reduced TRAF7 area percentage at E13.5 compared to the controls [38]. The aforementioned findings suggest that TRAF7 could be a critical factor in the normal development of the kidneys, and its deficiency could result in the development of CAKUT, specifically hypoplastic kidney, as evidenced by the current and previous evidence [20,38].

In P4, a statistically significant difference in TRAF7 expression was noted between the cortex and medulla of *yotari* mice relative to controls, with a tendency for higher expression in the medulla compared to the cortex. P4 denotes the end of phase 3 in the nephrogenesis of human samples, wherein we also demonstrated a greater area percentage of TRAF7 in the medulla relative to the cortex [20,42,43]. Differences in TRAF7 expression between the cortex and medulla likely reflect these kidney regions’ distinct functions and adaptive needs. Additionally, the previously mentioned potential stage-specific role of TRAF7 in kidney development may account for the general decrease in the percentage of TRAF7 surface area in *yotari* and wild-type mice. After the critical phase of nephrogenesis, TRAF7 expression decreases significantly. This pattern is also observed in other proteins and signaling pathways, such as NOTCH2, which exhibits stage-specific expression, is entirely silenced upon kidney maturation, and is also shown to be connected with the Reelin–Dab1 signaling pathway [38,54]. Notably, TRAF7 exhibited a distinct expression pattern compared to EDA2R and PCDH9 during kidney development. At postnatal day 14 (P14), particularly in *yotari* mutants, TRAF7 demonstrated strong punctate localization on the apical membranes of both distal and proximal convoluted tubules. This localization overlapped with Aqp2- and DBA-positive segments, indicating a potential role for TRAF7 in segment-specific epithelial differentiation or remodeling. Such a pattern aligns with TRAF7’s proposed functions in regulating apical–basal polarity and epithelial morphogenesis, suggesting that TRAF7 may contribute to nephron maturation by modulating epithelial specialization and membrane trafficking. These findings are consistent with previous observations that TRAF7 is involved in endothelial integrity and ciliogenesis, processes critical for normal kidney development and function [51].

A notable limitation of our study is the use of formalin-fixed paraffin-embedded (FFPE) tissue samples, which precluded the use of complementary techniques such as Western blotting or quantitative PCR for further validation of our findings. FFPE tissue preservation, while ideal for histological analysis, results in the degradation of RNA and proteins, making these techniques unsuitable for our study. As a result, the conclusions drawn from immunofluorescence analysis are based on percentage area quantification, which, while standardized and rigorously applied, could still be influenced by experimental artifacts inherent to the method. Despite this, our study is supported by extensive quality control measures, including the use of positive and negative controls, standardized imaging protocols, and expert validation of tissue morphology. Moreover, due to the limited research on these proteins, particularly in the context of nephrogenesis, it remains challenging to definitively determine their precise roles in the development of CAKUT. Nevertheless, our findings provide a solid foundation for future research in this field, which holds considerable scientific and clinical relevance. Subsequent studies should include not only more extensive tissue analyses but also complementary methods, such as urine analysis, to gain insight into key physiological parameters in mice, including the glomerular filtration rate. We believe that these safeguards ensure the reliability of our findings within the context of the available material and the specific focus on kidney development and disease mechanisms.

## 4. Materials and Methods

### 4.1. Ethics

The utilization of animals was authorized by the Guidelines for the Care and Use of Laboratory Animals at Shiga University of Medical Science. The experiment was carried out under the standards of the Declaration of Helsinki and obtained consent from the Ethical Committee of the University of Split School of Medicine (class: 003-08/23-03/0015, protocol code no.: 2181-198-03-04-23-0073, date of approval: 27 September 2023).

### 4.2. Generation of Dab1 Null Conventional Mice and Sample Acquisition

This investigation utilized *yotari* (*Dab1^−/−^*) mice, characterized as *Dab1* null conventional mutants, as previously delineated. C57BL/6N mice were reared and group-housed in regular polycarbonate cages (3–4 individuals, including at least a single member of each genotype) with unrestricted access to food and water in a temperature-regulated (23 ± 2 °C) environment. The photoperiod comprised 12 h of artificial illumination and 12 h of darkness.

The *yotari* mutation results from the replacement of two complete exons and part of an additional exon of the *Dab1* gene with a long interspersed nuclear element (L1) fragment. This genetic alteration leads to the production of mutated *Dab1* mRNA that does not translate into functional Dab1, effectively rendering the gene nonfunctional. Consequently, the *yotari* mouse is considered a functional null mutant for *Dab1* [55,56].

The subsequent PCR primers utilized for genotyping were *yotari*—GCCCTTCAG-CATCACCATGCT and CAGTGAGTACATATTGTGTGAGTTCC—and the wild-type of the *Dab1* locus—GCCCTTCAGCATCACCATGCT and CCTTGTTTCTTT-GCTTTAAGGCTGT [57,58].

The pregnant mice were euthanized on gestation days 13.5 (E13.5) and 15.5 (E15.5), and their embryos were retrieved. Additional cohorts of mice were euthanized on postnatal days 4 and 14 (P4 and P14). Three to four animals were utilized for each analyzed group. Initially, they were profoundly anesthetized with pentobarbital, thereafter undergoing transcardial perfusion with phosphate-buffered saline (PBS, pH 7.2), followed by 4% paraformaldehyde (PFA) in 0.1 M PBS. The kidneys were excised and individually fixed in 4% paraformaldehyde in 0.1 M PBS overnight for standard histological assessments, combining hematoxylin-eosin and immunofluorescence staining.

### 4.3. Immunofluorescence on Embryonic and Postnatal Mouse Renal Tissue

After fixation and drying of the tissue using graded ethanol solutions, the tissue was embedded in paraffin blocks and serially sectioned into five-micrometer-thick slices on a microtome (RM2125 RTS, Leica, Buffalo Grove, IL, USA), which were then mounted on adhesive microscope slides. Appropriate tissue preservation was confirmed through hematoxylin-eosin staining of every tenth segment.

Next, the immunofluorescence staining was performed, according to established protocols [45]. Following deparaffinization in xylene and rehydration in graded water-ethanol solutions, the resulting tissue samples were heated in a water steamer with 0.01 M citrate buffer (pH 6.0) for 30 min at 95 °C, allowing them to gradually cool to room temperature. After rinsing in 0.1 M PBS, a protein-blocking solution (ab64226, Abcam, Cambridge, UK) was applied for 30 min to prevent non-specific staining. Primary antibodies (Table 1) were applied to the sections and incubated overnight in a humidity chamber at room temperature. The next day, the sections were rinsed with PBS before a one-hour incubation with the appropriate secondary antibodies (Table 1). Finally, the samples were rinsed in PBS once more, and DAPI (4′,6-diamidino-2-phenylindole) staining was used to visualize the nuclei. The samples were then air-dried, mounted (Immuno-Mount, Thermo Shandon, Pittsburgh, PA, USA), and coverslipped.

To reduce non-specific background signals, isotype-matched controls and secondary-only samples were utilized. Isotype-matched controls involved replacing the primary antibody with a non-target-specific antibody of the same isotype, enabling the assessment of potential non-specific binding (Figure S1). Secondary-only controls, in which the primary antibody was omitted, were to detect any non-specific interactions of the secondary antibody (Figure S2). These used controls ensured that the observed fluorescence signals were specific to the target protein rather than artifacts such as residual paraffin or background noise. To verify the validity of the immunofluorescent staining, isotype-matched controls secondary-only controls and positive controls were utilized. Isotype-matched controls involved replacing the primary antibody with a non-target-specific antibody of the same isotype, enabling the assessment of potential non-specific binding. Secondary-only controls, in which the primary antibody was omitted, were used to detect any non-specific interactions of the secondary antibody. To validate antibody specificity, positive control staining was performed in E15.5 wild-type mouse tissues known to express the target proteins, confirming Eda2r in the skin, Pcdh9 in the choroid plexus, and Traf7 in the lungs (Figure S3). These controls ensured that the observed fluorescence signals were specific to the target protein rather than artifacts such as residual paraffin or background noise.

### 4.4. Data Acquisition and Analysis

Fluorescence imaging was conducted using an Olympus BX51 fluorescence microscope (Tokyo, Japan) equipped with a Nikon DS-Ri2 camera (Nikon Corporation, Tokyo, Japan) and operated with NIS-Elements F software (version 5.22.00). Images were acquired at 40× magnification with standardized gain, exposure, and white balance settings. The expressions of EDA2R, PCDH9, and TRAF7 were analyzed in a minimum of ten representative fields of view from embryonic and postnatal kidney specimens. In postnatal samples, at least ten images were obtained from the renal cortex and ten from the medulla. The analysis of the kidney medulla in the embryonic samples was excluded due to the limited number of available photomicrographs, which resulted from the extremely small tissue area. Additionally, distinguishing between the cortex and medulla in the embryonic kidney at this developmental stage was not feasible. Positive staining for EDA2R, PCDH9, and TRAF7 was observed as diffuse or punctate green fluorescent signals localized to distinct renal substructures.

Image processing was performed using ImageJ software 1.54g (National Institutes of Health, Bethesda, MD, USA) and Adobe Photoshop (Adobe, San Jose, CA, USA), following previously established and validated protocols [56,59]. To minimize fluorescence spillover, the red counter-signal was subtracted from the green fluorescence. Images were duplicated, and a median filter with an 8.0-pixel radius was applied in a single iteration. The positive signal was isolated by subtracting the filtered images from the originals. The processed images were subsequently converted to an 8-bit format and subjected to threshold adjustment using the triangle thresholding algorithm. Fluorescence quantification was performed using the “Analyze Particles” function to determine the percentage area of fluorescence. To summarize, the “area percentage” refers to the proportion of the image occupied by the positive fluorescent signal, calculated as the number of fluorescent pixels above a defined threshold divided by the total number of pixels in the image. The results were averaged per examined group.

### 4.5. Statistical Analyses

Statistical analyses were performed using GraphPad Prism software (version 9.0.0, GraphPad Software, San Diego, CA, USA). A two-way analysis of variance (ANOVA) followed by Tukey’s multiple comparison test was used to assess differences in the area percentage of EDA2R, PCDH9, and TRAF7-positive cells across various developmental stages (E13.5, E15.5, P4, and P14). Regarding the postnatal developmental time points (P4 and P14), we averaged the area percentage between the cortex and medulla. As previously mentioned, such regional distinction was not feasible in the embryonic samples due to the relatively small size of the kidney tissue and the limited ability to clearly separate the cortex and medulla at that developmental stage.

Additionally, differences in immunoexpression between the renal cortex and medulla were examined at postnatal days 4 and 14 between the observed phenotypes. The data were presented as the mean ± standard deviation (SD), with statistical significance at *p* < 0.05.

Graphs were generated using GraphPad Prism software. The figure plates were assembled using Adobe Photoshop (version 21.0.2). Aquaporin 1 (Aqp1) and Aquaporin 2 (Aqp2), which are well-characterized water channel proteins with essential roles in renal physiology, particularly in regulating water reabsorption and urine concentration, as well as Dolichos biflorus agglutinin (DBA), Lotus tetragonolobus lectin (LTL), and carbohydrate-binding lectins were not the focus of the investigation but were used solely as compartment-specific markers to identify and visualize distinct nephron segments to contextualize the spatial localization of our proteins of interest (EDA2R, PCDH9, and TRAF7). Specifically, LTL was used to identify differentiated proximal tubules, DBA to label developing collecting ducts and distal tubules in the postnatal kidney cortex, Aqp1 to mark the apical and basolateral membranes of proximal tubule cells, and Aqp2 as a marker of principal cells in developing collecting ducts and ureteric bud derivatives.

Microphotographs underwent background removal and contrast enhancement to enhance visibility and clarity. For illustration purposes, immunofluorescence images shown in the figures were selected to highlight clear examples of colocalization between the protein of interest (e.g., TRAF7) and specific nephron segment markers (LTL, DBA, Aqp1, and Aqp2). These images are not intended to represent average expression levels but to demonstrate spatial localization patterns. In cases where average expression was low (e.g., <1%), regions with slightly higher localized signals were chosen to allow the visual confirmation of marker overlap.

## 5. Conclusions

EDA2R, PCDH9, and TRAF7, the CAKUT candidate gene proteins, demonstrated the highest area percentage at E13.5. This percentage decreased as postnatal stages progressed, indicating the potential involvement of these proteins in the development of CAKUT. The most important statistically significant difference was observed in TRAF7, whose area percentage was diminished in *yotari* compared to wild-type mice, suggesting an association with Reelin–Dab1 signaling and a possibly pivotal involvement in the pathogenesis of CAKUT, as also shown by our previous findings. Ultimately, these proteins, particularly TRAF7, may serve as possible novel biomarkers for identifying CAKUT; however, additional research is required.

## Figures and Tables

**Table 1:** Antibodies used for immunofluorescence.

Antibodies		Catalog Number	Host	Dilution	Source
Primary	Anti-EDA2R/XEDAR antibody	ab203667	Rabbit	1:120	Abcam (Cambridge, UK)
	PCDH9 Polyclonal antibody	25090-1-AP	Rabbit	1:200	Proteintech Group, Inc. (Rosemont, IL, USA)
	TRAF7 Polyclonal antibody	11780-1-AP	Rabbit	1:50	Proteintech Group, Inc. (Rosemont, IL, USA)
	Aquaporin 1/Aqp1 A antibody (B-11)	sc-25287	Mouse	1:50	Santa Cruz Biotechnology (Dallas, TX, USA)
	Aquaporin 2/Aqp2 antibody (E-2)	sc-515770	Mouse	1:50	Santa Cruz Biotechnology (Dallas, TX, USA)
Lectins	Fluorescein labeled Lotus tetragonolobus lectin (LTL)	FL-1321	N/A	1:400	Vector Laboratories Ltd., Peterborough, UK
	Fluorescein labeled Dolichos biflorus agglutinin (DBA)	FL-1031	N/A	1:400	Vector Laboratories Ltd., Peterborough, UK
Secondary	Rhodamine Red™-X (RRX) AffiniPure™ Donkey Anti-Mouse IgG (H + L)	715-295-151	Donkey	1:400	Jackson Immuno Research Laboratories, Inc. (Baltimore, PA, USA)
	Alexa Fluor^®^ 488 AffiniPure™ Donkey Anti-Mouse IgG (H + L)	715-545-150	Donkey	1:300	Jackson Immuno Research Laboratories, Inc., Baltimore, PA, USA

## Data Availability

All data and materials are available upon request.

## Supplementary Materials

The following supporting information can be downloaded at https://www.mdpi.com/article/10.3390/ijms26136421/s1.

## Abbreviations

The following abbreviations are used in this manuscript:

CAKUT	congenital anomalies of the kidney and urinary tract
EUROCAT	European Registration of Congenital Anomalies and Twins
FFPE	formalin-fixed paraffin-embedded
NHS	National Health Service
KRT	kidney replacement therapy
ESRD	end-stage renal disease
CNV	copy number variations
EDA2R	Ectodysplasin A2 Receptor
PCDH9	Protocadherin 9
TRAF7	TNF receptor-associated factor 7
EMT	epithelial-to-mesenchymal transition
DCT	distal convoluted tubules
PCT	proximal convoluted tubule
E	embryonic day
P	postnatal day
