# Supplementary material for: Spatial and Temporal Expression Patterns of EDA2R, PCDH9, and TRAF7 in Yotari (Dab1−/−) Mice: Implicationsfor Understanding CAKUT Pathogenesis
Source: Int J Mol Sci. 2025 Jul 3;26(13):6421. doi: 10.3390/ijms26136421 (PMC12249912; doi:10.3390/ijms26136421)
Supplement: Supplementary file 1 [file ijms-26-06421-s001.zip › ijms-3700096-supplementary.pdf]

International Journal of
Molecular Sciences

MDPI

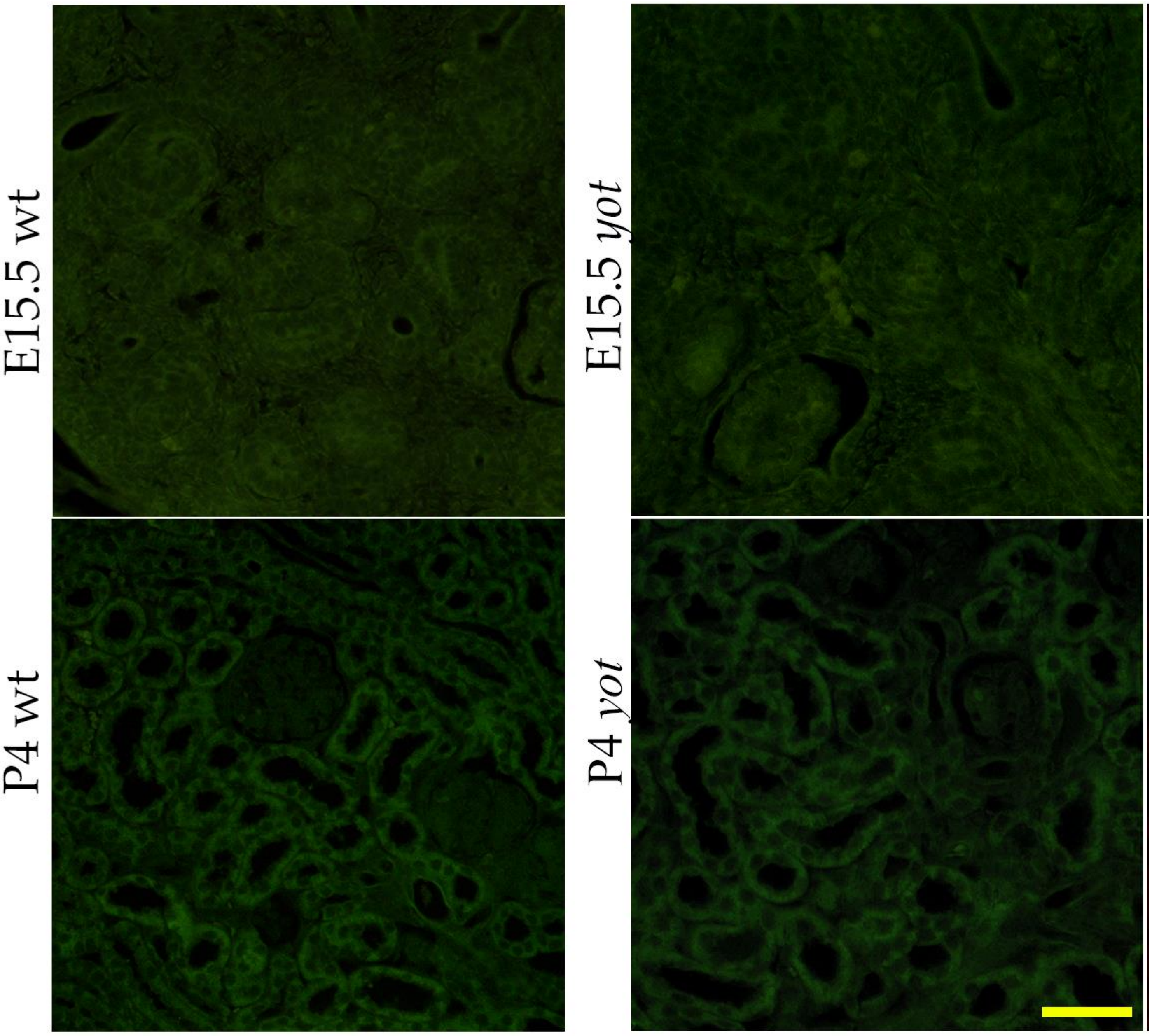

Figure S1. Isotype-matched control staining of embryonic day 15.5 (E15.5) and postnatal day 4 (P4) wild-type (wt) and *yotari* (*yot*) mouse kidneys. Primary antibodies were replaced with isotype-matched control immunoglobulins, followed by secondary antibody incubation using the standard immunofluorescence protocol. Images were taken at 40× magnification. Scale bar: 50 µm (applies to all images).

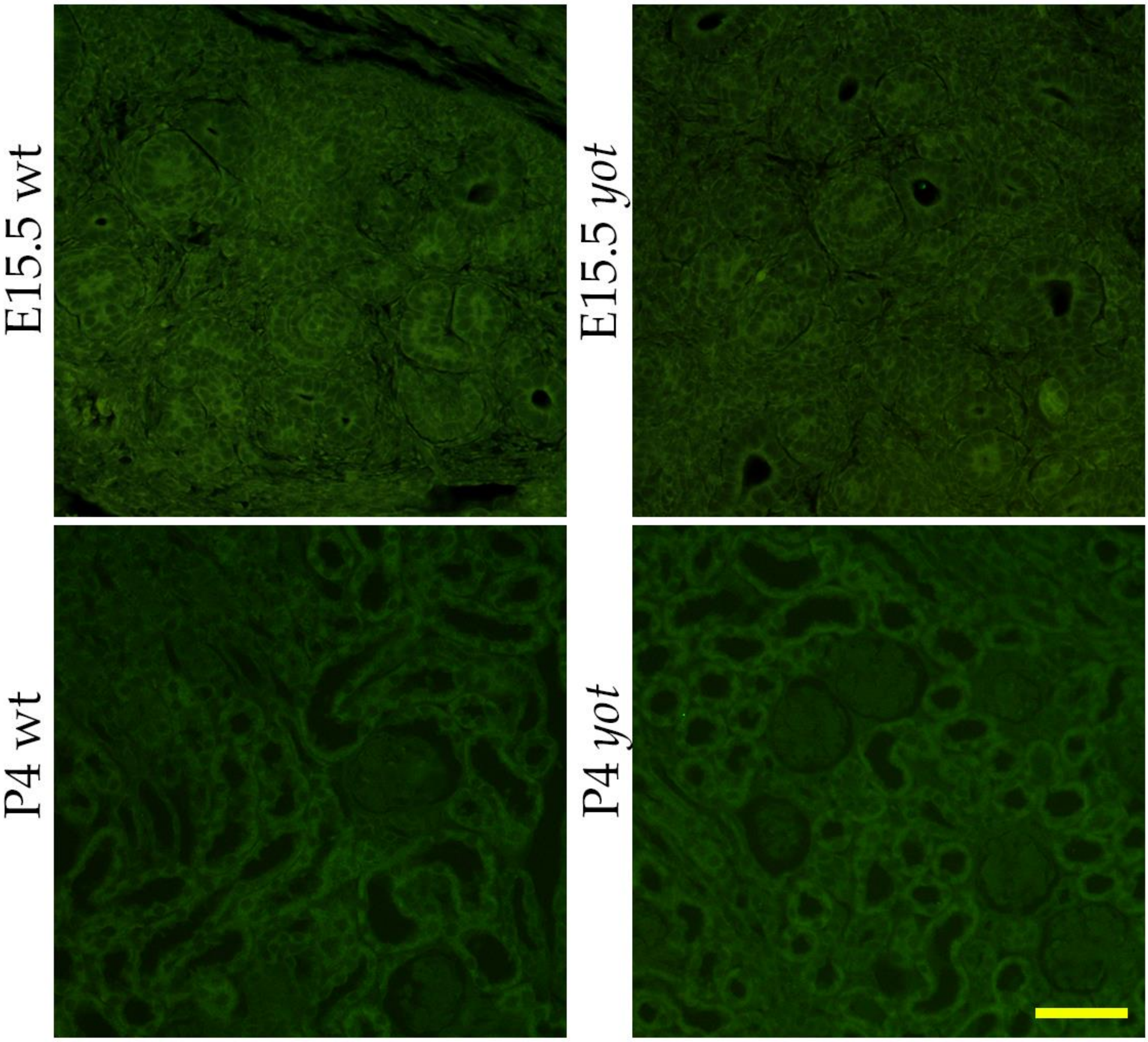

Figure S2. Negative control staining of embryonic day 15.5 (E15.5) and postnatal day 4 (P4) wild-type (wt) and *yotari* (*yot*) mouse kidneys. Primary antibodies were omitted from the immunofluorescence protocol, and only secondary antibodies were applied to the sections. Images were taken at 40× magnification. Scale bar: 50 μm (applies to all images).

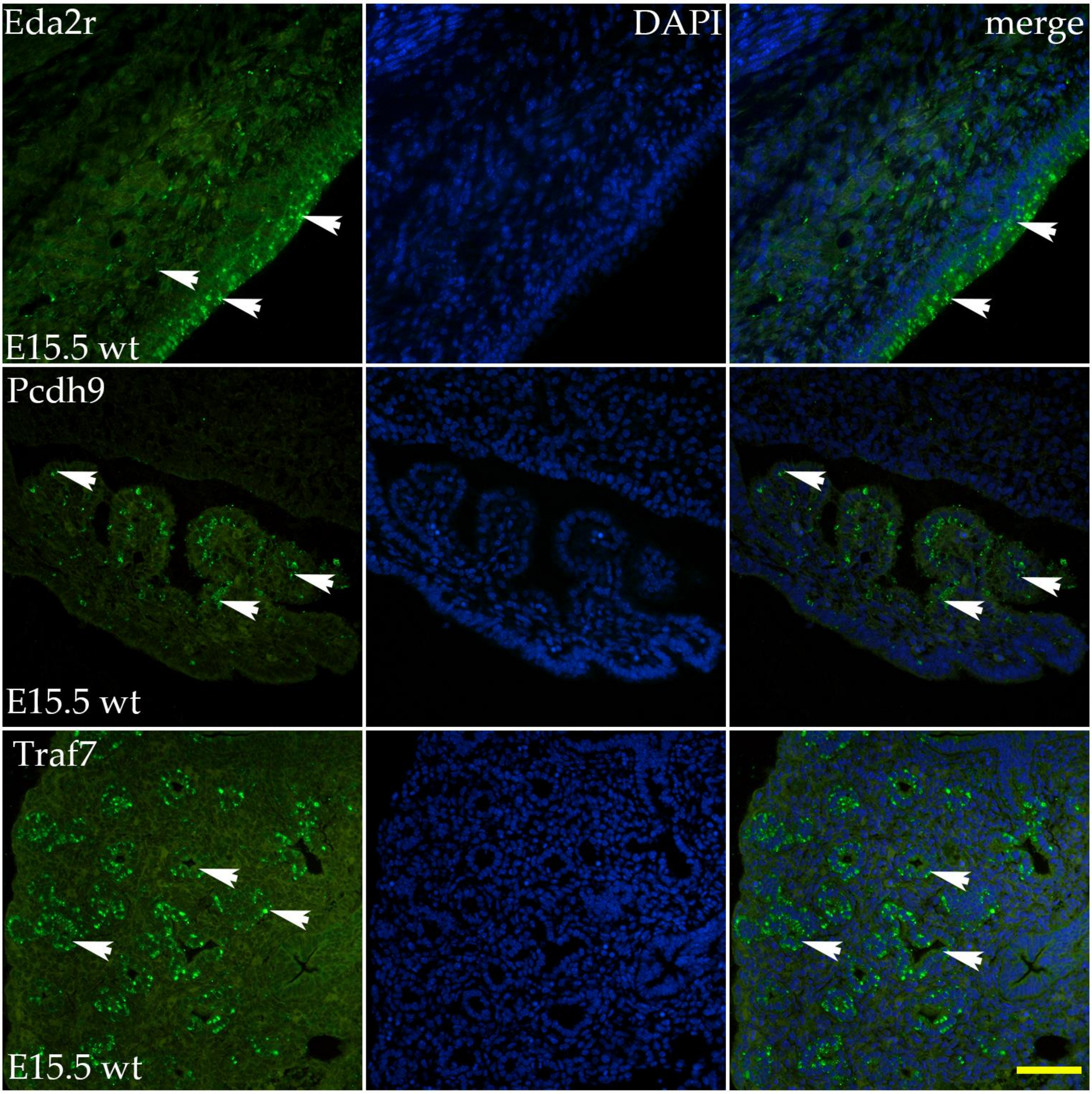

Figure S3. Positive control staining for Eda2r, Pcdh9, and Traf7 in tissues known to express these proteins in embryonic day 15.5 (E15.5) wild-type (wt) mice. Eda2r expression (white arrows) was confirmed in the skin, Pcdh9 in the choroid plexus, and Traf7 in the lungs. Images were taken at 40× magnification. Scale bar: 50 µm (applies to all images).
